# Energy Efficiency in Turning: A Comparative Analysis of Screw Drive and Linear Drive CNC Machine Tools

**DOI:** 10.3390/ma18010075

**Published:** 2024-12-27

**Authors:** Agnieszka Terelak-Tymczyna, Krzysztof Marchelek, Ryszard Daniel Ziętek, Paweł Frankowski, Agata Zubkiewicz, Karol Miądlicki

**Affiliations:** 1 Faculty of Mechanical Engineering and Mechatronics, West Pomeranian University of Technology in Szczecin, al. Piastów 19, 70-310 Szczecin, Poland; aterelak@zut.edu.pl (A.T.-T.); krzysztof.marchelek@zut.edu.pl (K.M.); r.zietek@pm.szczecin.pl (R.D.Z.); 2 Faculty of Computer Science and Telecommunications, Maritime University of Szczecin, Wały Chrobrego 1-2, 70-500 Szczecin, Poland; p.frankowski@pm.szczecin.pl; 3 Institute of Mathematics, Physics and Chemistry, Maritime University of Szczecin, Wały Chrobrego 1-2, 70-500 Szczecin, Poland; a.zubkiewicz@pm.szczecin.pl; 4 Faculty of Mechatronics and Electrical Engineering, Maritime University of Szczecin, Willowa 2, 71-650 Szczecin, Poland

**Keywords:** CNC machine tools, energy efficiency, screw drive, linear drive, reactive power compensation, turning

## Abstract

This paper presents a comparative analysis of the energy efficiency of screw drive and linear drive CNC machine tools in turning operations. Two CNC lathes were investigated, one equipped with screw drives and the other with linear drives, during the turning of specially prepared parts. The research examines active and reactive energy consumption, offering insights into the energy efficiency of different drive technologies. The analysis indicates that lathes with linear drives exhibited a higher reactive power consumption (8 kVar) during idle operation in comparison to those with screw drives (1.2 kVar). However, both drive systems demonstrated comparable potential for reducing reactive power consumption through implementing compensation techniques, with a reduction in reactive power consumption of nearly 70%. For both drive systems, the reduction in power use with compensation was at the level of 23–30% for screw drives and 36–47% for linear drives. The study highlights the importance of considering both active and reactive energy in evaluating the energy efficiency of machine tools. The findings contribute to a deeper understanding of energy consumption in turning processes, aiding in the selection and optimization of drive systems for improved sustainability in manufacturing. Future research should explore tool wear impacts, machine-specific energy optimization, and AI-driven solutions for real-time energy management.

## 1. Introduction

**Figure 1:**
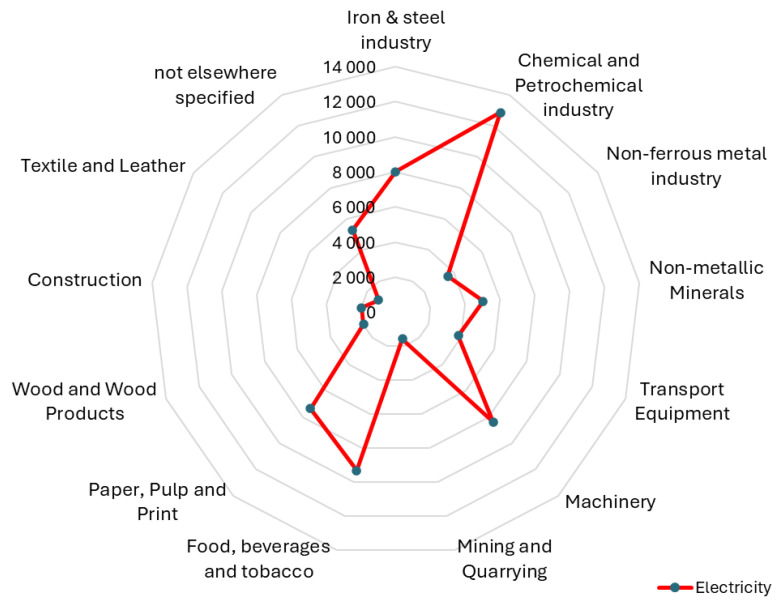
Electricity consumption by industry sector [2].

Electricity represents one of the fundamental forms of energy utilized within enterprise operations. It serves as the primary energy source for machinery, drives the processes that enable manufacturing, and is essential for the continuous operation of factories. However, this reliance on electricity has economic and environmental associated costs [1]. The machining industry, especially energy-intensive processes such as turning, milling, and grinding, is a notable electricity consumer, frequently ranking third only to the chemical and food, beverages, and tobacco industries in terms of energy intensity (Figure 1) [2]. The manufacturing industry faces mounting pressure to reduce its environmental impact and enhance sustainability [3]. A principal objective is to improve energy efficiency, which strives to reduce energy consumption without sacrificing productivity or quality [4]. In turning processes, where material is removed from a rotating workpiece using a cutting tool, energy consumption is a significant factor affecting both operating costs and environmental footprint [5]. As defined in EN 16247-1: Energy audits–Part 1 [6], energy efficiency is a measure of the ratio of the result of an organization’s activities, products, services, or energy to the energy used for input. The term “general requirements” refers to the ratio, or other quantitative relationship, between an organization’s activities, products, services, or energy consumption and the energy used as an input. Examples include the ratio of energy required to energy used, the ratio of output to input elements, and the ratio of theoretical energy used for work to energy used for work. It is essential that both input and output elements are clearly defined in terms of quantity and quality and that they are measurable.

**Figure 2:**
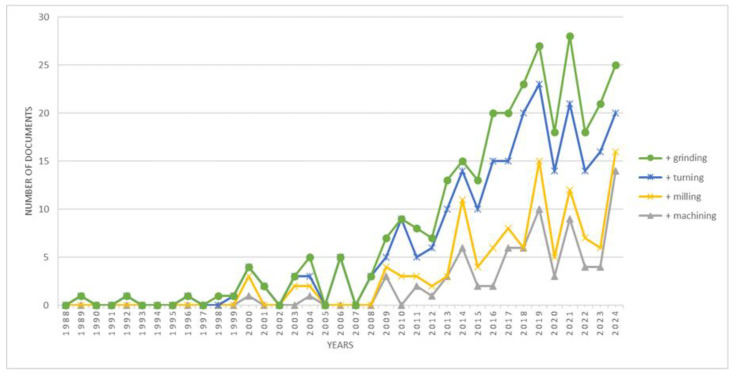
Number of publications considering reactive power in machining, grinding, turning, and milling processes [Scopus database].

New technologies are transforming the manufacturing industry, offering significant improvements in speed and efficiency [7,8]. The results of studies and simulations presented in the literature refer to the analysis of active energy without considering the amount of reactive energy consumed [9]. An analysis of publications in the Scopus database related to the analysis of reactive energy shows that the number of publications related to this topic has been growing since 2008. Despite the growing interest in reactive energy, there have been less than one hundred publications relating to machining over the past 30 years, as detailed in Figure 2. The growing number of publications is undoubtedly associated with the development of information systems and devices for remote recording and management of the electrical network in manufacturing plants, as well as normative and legal regulations in the energy efficiency of manufacturing plants. As early as March 2007, the European Council’s conclusions highlighted the need to increase energy efficiency in the European Union to achieve 20% savings in primary energy consumption compared to the projections Directive (EU) 2023/1791 [10]. A significant contribution to the growing interest in energy efficiency in appliances, including attention to reactive energy, is undoubtedly due to the 2009 publication of the British Standard BS EN 16001:2009 [11], which in 2011 was replaced by the international standard ISO 50001 [12]. Another impetus for increased interest in energy efficiency and reactive energy consumed by equipment was the entry into force of Directive 2012/27/EU of the European Parliament and of the Council of 25 October 2012 on energy efficiency, amending Directives 2009/125/EC and 2010/30/EU and repealing Directives 2004/8/EC and 2006/32/EC, regulating energy management and energy efficiency regulations of the European Union member states. The relevance of the topic is confirmed by the update of the energy efficiency regulation in 2023. (Directive (EU) 2023/1791).

**Figure 3:**
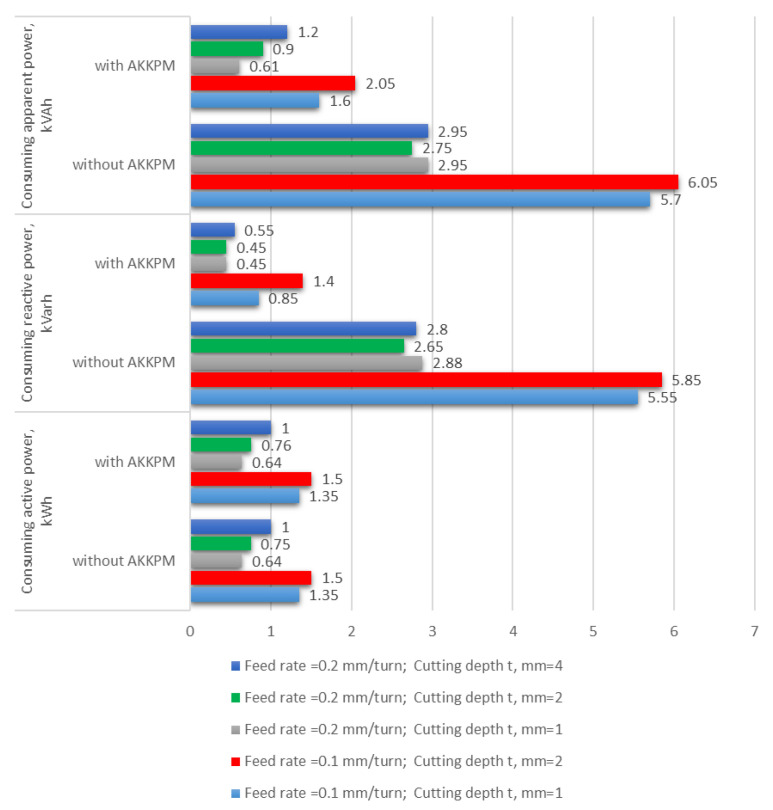
Electricity consumption for machine tool with and without compensation [2].

Analysis of the reactive energy consumed by machine tools, along with research and development of industrial electronics/automatics components, has so far been handled by a research team established at Siemens. This team has developed a machine tool control with visualization for monitoring the energy parameters of a machine tool with the possibility of reducing energy consumption during machine shutdowns. In addition, capacitors can be used with the latest machine tool control for reactive power compensation. Using the device, according to the information provided by the manufacturer, allows one to achieve cosϕ = 1. The issue of reactive energy used by machine tools was also dealt with by Zmieva [13], who indicated that by using individual compensation on the machine tool, we could achieve savings in the total energy consumed by the machine tool of 59–72%, as shown in Figure 3.

The research team of Kianinejad et al. [14] also considered reactive energy consumption, which compared the energy consumption of two milling machines. Various methods are employed, including capacitor banks and synchronous machines [15,16]. Synchronous compensation offers advantages in mitigating harmonic distortion compared to capacitive compensation [17]. Active energy filters can compensate for harmonic distortion in motor soft starters [18]. The conservative power theory has been applied for reactive and harmonic compensation in shunt compensators [19]. Proper reactive power compensation can reduce voltage fluctuations and line losses and improve power factors in industrial settings [20]. In synchronous machines, active compensation of unbalanced magnetic pull can eliminate damper bar currents and stator parallel circuit circulating currents, potentially increasing electromagnetic efficiency [21]. Reactive power compensation can save energy and reduce CO_2_ emissions [22]. Reactive power compensation is crucial for improving energy efficiency and power quality in CNC control systems, particularly those with nonlinear loads and semiconductor converters [18]. Various methods exist for reactive power compensation, including active energy filters, capacitor banks, and advanced control strategies [23]. In CNC machine tools, machine learning techniques can be applied for precise and cost-effective thermal error self-compensation [24]. For power systems with semiconductor converters, multi-pulse circuits, and filter-compensation devices can reduce harmonic distortion and improve power quality [25]. In inductive power transfer systems, different compensation topologies using capacitors are employed to enhance efficiency [26]. Recent research has also explored the use of regenerative controlled-speed synchronous motor drives for reactive power compensation in industrial applications [27].

### 1.1. Energy in Machining

Turning, as a foundational machining process used across various industries, offers a prime opportunity for energy optimization. Analyzing energy consumption in turning is crucial for reducing costs, minimizing environmental impact, gaining a competitive advantage, and conserving resources. The literature on energy intensity in machining often explores two main aspects: the machine tool and the machining process [28,29]. Machine tool-related research delves into energy consumed by various components, including idle consumption, drive system and motor efficiency, auxiliary systems, and machine design. Machining process-related research focuses on the energy used during cutting, examining factors like cutting parameters, tool-workpiece interaction, the material removal rate, cutting fluids, and process optimization. These two aspects are interconnected, as the efficiency of the machine tool influences the energy required for the machining process and vice versa. Analyzing electricity consumption by individual drives of machine tools in turning operations is critical for enhancing energy efficiency and promoting sustainability in manufacturing processes. Various studies have explored the energy consumption patterns of machine tools, focusing on different components and operational phases. According to an investigation conducted by Li et al., the most significant contributors to the machine tool’s energy consumption are the cooling and lubrication system (31%) and the hydraulic system (27%). To a lesser extent, energy consumption is affected by auxiliary systems (19%) and servo drives (17%) [30]. The authors also investigate fixed-energy consumption in machine tools, which is crucial for maintaining machine readiness. They argue that understanding the energy dynamics during standby modes is often overlooked, yet it plays a significant role in the total energy footprint of machine tools. This aspect is particularly relevant when analyzing the electricity consumption of individual drives, as standby energy can constitute a substantial portion of total consumption. Kianinejad et al. emphasize the necessity of reliable energy consumption data under realistic machining conditions to assess the efficiency of outdated cutting machines. Their research highlights how different process parameters, machining materials, and the ratio of primary to secondary time significantly influence energy consumption and machining efficiency [14]. This understanding is crucial for identifying improvement potentials in older machines, which often exhibit higher energy consumption than newer models. Vijayaraghavan and Dornfeld provide insights into the automated energy monitoring of machine tools, where they classify power consumption into productive and non-productive periods. Their empirical analysis reveals that various machine tool components have distinct power requirements, which can be systematically monitored to identify inefficiencies [31]. This classification is essential for understanding how individual drives contribute to overall electricity consumption during turning operations. Behrendt et al. outline the development of an energy consumption monitoring procedure specifically for machine tools, which can facilitate identifying energy-saving opportunities [32]. Their work underscores the importance of systematic monitoring in understanding the operational energy demands of different machine components, including the drives used in turning operations. Researchers in [33] discuss strategies for improving energy efficiency in machine tools, emphasizing the need for comprehensive studies encompassing all operational phases, including setup, machining, and idle times. This holistic approach is vital for accurately assessing the energy consumption of individual drives during turning processes. Lastly, the investigation by Lv et al. into spindle acceleration energy consumption highlights the specific energy demands associated with machine tool operations [34]. Their findings suggest that optimizing spindle acceleration can lead to significant reductions in energy consumption, which is particularly relevant when analyzing the drives responsible for these functions.

Another approach to the energy usage problem is to examine the part machining process in terms of energy consumption. In this case, the following issues should be considered: the selection of machining parameters, the planning of the machining process, or even the modeling machining process. The selection of machining parameters, planning the process, and modeling machining processes are critical components in optimizing manufacturing efficiency and sustainability. Various studies have highlighted the importance of these factors in achieving desired outcomes such as reduced energy consumption, improved surface finish, and enhanced material removal rates. The selection of machining parameters, including cutting speed, feed rate, and depth of cut, is essential for optimizing performance in machining operations. Datta and Majumder emphasize that optimizing these parameters can lead to improved productivity, which is characterized by minimal surface roughness and maximum material removal rates while minimizing tool wear [35]. Similarly, Jia et al. proposed a multi-objective parameter optimization method for CNC milling that incorporates energy modeling and complex constraints, demonstrating the intricate relationship between cutting parameters and performance metrics [36]. This approach enhances machining efficiency and aligns with sustainable manufacturing practices by reducing energy consumption during the milling process.

Regarding process planning, energy efficiency has emerged as a significant consideration. Newman et al. present a framework that integrates energy consumption into CNC machining process planning objectives, indicating that energy-efficient machining can substantially lower industrial energy consumption [37]. Similar research investigations were conducted by Liu et al., who discuss determining energy-efficient cutting parameters while ensuring specified machining accuracy, thus highlighting the dual focus on quality and energy efficiency in process planning [38]. The need for a balanced approach is echoed by Wang et al., who note that minimizing machining time can inadvertently increase total energy consumption, underscoring the complexity of process planning in machining [39]. Modeling the machining process is another vital aspect that facilitates the optimization of parameters. Zhang et al. introduced a process parameters optimization method specifically for multi-pass dry milling, which aims to achieve high efficiency with low energy consumption and carbon emissions [40]. These results align with Yi et al.’s findings, which explored the effects of various cutting fluid types on energy consumption and tool life, proposing a framework that breaks down machining processes into manageable activities for better energy management [41]. Furthermore, empirical models developed by Velchev et al. for specific energy consumption during turning provide a quantitative basis for optimizing cutting parameters to minimize energy use [42].

In summary, the collective insights from these studies illustrate the complexity of electricity consumption in machine tools, particularly during turning operations. By examining various components, operational phases, and the influence of different parameters, researchers can develop targeted strategies to enhance energy efficiency and reduce the environmental impact of manufacturing processes. The relation between the selection of machining parameters, process planning, and modeling is crucial for enhancing machining efficiency and sustainability. The integration of energy considerations into these processes not only leads to improved operational performance but also supports broader environmental goals in manufacturing.

### 1.2. Drive Types and Commercial CNC Systems

Linear drives are increasingly being adopted in machine tools because they can accelerate and decelerate rapidly, leading to faster production rates and reduced cycle times [43]. This increased speed translates to higher throughput and quicker turnaround times, which is crucial in today’s competitive manufacturing landscape. However, this boost in productivity often comes with a trade-off in energy consumption. While linear drives offer advantages in terms of dynamic performance, they can sometimes require more energy compared to traditional screw drives. Linear motors directly generate linear motion and may have higher power demands, especially during rapid acceleration and deceleration phases. In contrast, screw drives, which convert rotary motion to linear motion via a ballscrew, can be more energy-efficient in certain operating conditions. The energy consumption of both linear and screw drives is influenced by several factors, including the mass of the moving components, the required acceleration and deceleration rates, and the efficiency of the motor and drive system. Furthermore, the specific application and operating conditions play a significant role. For instance, linear drives might consume more energy in applications involving frequent starts and stops or high-speed movements. Conversely, in applications with smoother motion profiles and less demanding acceleration requirements, screw drives might prove to be more energy efficient. Therefore, it is essential to analyze and optimize energy consumption in turning processes, with a particular focus on the efficiency of different drive systems, such as screw drives and linear drives. A comprehensive analysis of both systems’ energy requirements and performance characteristics will pave the way for a more sustainable and cost-effective machining industry.

**Figure 4:**
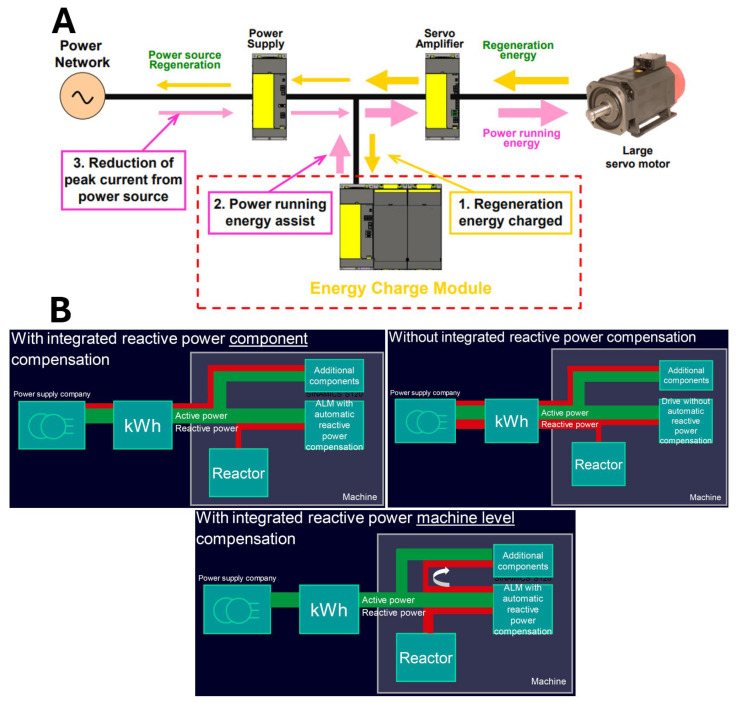
Schematic of a braking energy recovery unit proposed by Fanuc (**A**) and Siemens (**B**). Based on [44,46].

**Figure 5:**
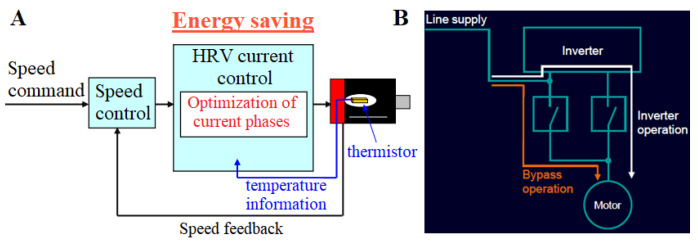
Temperature-based motor current phase control scheme proposed by Fanuc (**A**) and inverter bypass operation proposed by Siemens (**B**). Based on [47,48].

When considering the current state of knowledge in the energy efficiency of machinery and equipment, one should not look only at the amount of active energy consumed according to the definition of sustainability. Sustainable development of the economy is based on the consideration of low emissivity and reduction in environmental pollution in such a way that its condition will not deteriorate for future generations. To achieve a state of low emissivity of machinery and equipment, we should pay attention not only to direct emissions emitted during the operation of the equipment or machine but also to indirect emissions related to the entire life cycle. Indirect emissions during operation are associated with consuming materials, water, compressed air, and electricity. When analyzing the electricity consumption of a machine or device, we should not only consider that part of the energy that is consumed for work, i.e., active electricity. Equally important from both an economic and energy point of view is reactive electricity. The latter is often overlooked in various types of analysis. Older machine tools were primarily mechanical, with less emphasis on complex electronics and high-power motors—this is a simple fact. Reactive power consumption was not a significant issue, and the cost-benefit of compensation systems was not as compelling. Modern machine tools depend on powerful electric motors, variable frequency drives, and sophisticated control systems. These components draw significant reactive power, inevitably leading to inefficiencies and higher energy costs. New CNC control systems have integrated sensors and monitoring systems that can accurately measure reactive power consumption in real time, and these data are crucial for effective reactive power compensation. These systems are mounted in the latest machines with advanced measuring devices and CNC controls. Modern CNC controls can dynamically adjust the operation of reactive power compensation devices (like capacitor banks) to optimize performance based on the machine’s operating conditions. Energy compensation in manufacturing machinery is a fundamental aspect that requires more discussion. When implemented using advanced technologies, it fits perfectly into the Industry 5.0 paradigm, creating more environmentally friendly solutions. The drive component is the first area in which energy consumption can be reduced. The most effective solutions are energy recovery systems during servo braking [44,45] (Figure 4) and motor current phase control solutions that adapt based on motor temperature information (Figure 5A).

An energy storage module is used to realize energy recovery in such systems. Synchronous and asynchronous motors also reduce electricity consumption with very high efficiency [47]. Energy consumption is compensated through reactive power compensation, which provides a power factor of 1 [45]. Bypass operation (Figure 5B) is the best way to reduce the energy consumption of the drive component. The above method switches the power source from the inverter to direct from the grid, which minimizes the losses associated with inverter operation. You can also minimize energy consumption by using auxiliary equipment in the machine tool to put it into hibernation mode.

Another aspect related to energy compensation is information tools. Through a graphical user interface implemented on the operator panel, they provide information on electricity consumption and CO_2_ emissions. The values of individual indicators and the history of their changes allow for appropriate diagnostics, the results of which can be used to optimize the production process. Energy compensation is related to both the machine itself and the machining process. Historically, the machining process for complex parts often requires finding the optimal parameters by performing many trials. This approach carried high energy and material losses. A currently developed approach is digital twin technology [45,48,49], which is an accurate digital representation of the actual machine. That representation makes it possible to run tests and select the appropriate parameters offline on a computer, significantly reducing energy and material costs. Another aspect that reduces energy consumption is using hybrid machining centers that allow both cavity and additive machining. This approach offers the possibility of remanufacturing old parts, thereby reducing the potential amount of energy associated with, among other things, the manufacture of the prepreg, the eventual heat, and thermo-chemical treatment process, which are part of the manufacturing process of a new part. Compensation is also realized by maximizing the machining speed. A higher speed will be associated with increased power consumption, but the amount of energy consumed will be less due to the shorter operating time of the auxiliary units.

## 2. Materials and Methods

This study addresses a significant gap in the existing body of knowledge regarding the energy consumption characteristics of different machine tool drive systems, particularly in turning operations. While previous research has extensively explored energy efficiency in various machining processes, including CNC machining and milling, there remains a lack of focused comparative analysis specifically targeting screw drive versus linear drive systems in turning applications. One of the primary gaps identified is the insufficient understanding of how different drive mechanisms influence energy consumption during turning operations [50,51,52,53,54]. However, this research does not delve into the comparative aspects of different drive systems, which were crucial for optimizing energy use in turning. The authors discuss the energy efficiency of cutting machine tools and identify potential improvements, yet their analysis does not extend to a direct comparison between different drive technologies. This lack of comparative analysis is in other works, where researchers focus on parameter optimization for sustainable manufacturing but do not address the specific energy implications of different drive systems in turning. This work expands the knowledge base by providing a comparative analysis of energy efficiency between screw drive and linear drive machine tools in turning applications. This analysis is essential for advancing understanding of how different drive technologies can be leveraged to enhance energy efficiency in manufacturing processes. The purpose of this article is to present the results of the analysis of electricity consumption divided into active and reactive energy used by two machine tools: one with a screw drive (DMG Mori Seiki Co, Pleszew, Poland, CNC DMG CTX 310 eco) and the other with linear drives (DMG Mori Seiki Co, Bielefeld, Germany, CNC CTX 420 Linear). Unlike the studies presented in the literature, the results presented in this article show the effect of using drives with different energy characteristics in machine tools on energy efficiency, considering the consumption of both active and reactive energy. The research includes turning two specially prepared parts on the machines mentioned above and an energy analysis of the entire machining cycle.

### 2.1. Energy Calculations

Analyzing the energy consumed by machine tools, it is necessary to consider that the device is connected to an AC electrical system. As a result, in machine tool drives, the current undergoes a phase shift relative to the voltage, resulting in the consumption of apparent energy (reactive and active). Apparent power *S* (1) is expressed in kilovolt-ampere-hours (kVAh) and corresponds to apparent power *S* (kVA) (Equation (1)).

(1) $$S=\sqrt{{\left(P\right)}^{2}+{\left(Q\right)}^{2}}$$

Herein, we have the following:

*S*—apparent power,

*P*—active power,

*Q*—reactive power.

Active energy is expressed in kilowatt-hours (kWh). It is used in front of the machine tool by converting it into mechanical work and corresponds to the active power *P* (kW) expressed by the Equations (2) (three-phase network) and (3) (single-phase network):

(2) $$P=\sqrt{3}\cdot U\cdot I\cdot \cos\varphi$$

(3) P=U·I·cos⁡φ

Herein, we have the following:

*P*—active power,

*U*—voltage,

*I*—current.

Reactive energy is expressed in kVArh. It is the amount of power lost in the system multiplied by the number of intervals per hour. It can come in two types: capacitive and inductive reactive energy. In machine tools, this energy is consumed in the windings of motors to generate a magnetic field, without which these devices could not function. Reactive energy corresponds to reactive power *Q* (kVAr), which is expressed by Equations (4) (three-phase network) and (5) (single-phase network):

(4) $$Q=\sqrt{3}\cdot U\cdot I\cdot \sin\varphi$$

(5) Q=U·I·sin⁡φ

Herein, we have the following:

*Q*—reactive power,

*U*—voltage,

*I*—current.

Because of the lack of reactive energy to perform work by an appliance, this energy is often called “unproductive” for the user. For this reason, it is frequently overlooked when conducting energy efficiency analyses for appliances. The level of reactive energy consumed by an appliance can be calculated from the so-called power factor *cosφ,* which is equal to the ratio of active power *P* to apparent power *S*. The magnitude of the power factor is variable and depends on the location of the installed equipment and the way it is used. Energy suppliers use the *tg φ* factor in the electricity supply contract, which represents the ratio of reactive energy *E(Q)* (kVArh) to active energy *E(P)* (kWh) consumed during the same period. The number of charges for electricity consumed depends on this relationship. In distribution networks, the charge for the use of reactive energy is described by Equation (6):

(6) $$Ob=k\cdot C_{rk}\cdot \left(\sqrt{\frac{1+\tan^{2}\varphi }{1+\tan^{2}\varphi_{0}}}\right)\cdot E(P)$$

Herein, we have the following:

k—the price multiplier *Crk* set in the tariff,

$C_{rk}$—the average price of electricity in the competitive market,

$tg\;\varphi_{0}$—contractual power factor,

tg φ—power factor determined as the quotient of reactive energy *Q* and active energy *P* consumed by the customer during the billing period.

$E\left(P\right)$—active energy consumed in each billing period 24 h a day or in the time zone in which reactive energy consumption is controlled in MWh or kWh.

As defined by Directive (EU) 2023/1791 of the European Parliament and of the Council of 13 September 2023 on energy efficiency and amending Regulation (EU) 2023/955, all technological, behavioral, and economic changes improve energy efficiency. By using the power factor control system, several benefits can be achieved [55,56]:

- Reduction in energy charges;
- Reduction in ordered power;
- Reduction in active power losses in conductors by reducing the flow of electricity in the system;
- Improvement of the voltage value at the end of the line.

**Figure 6:**
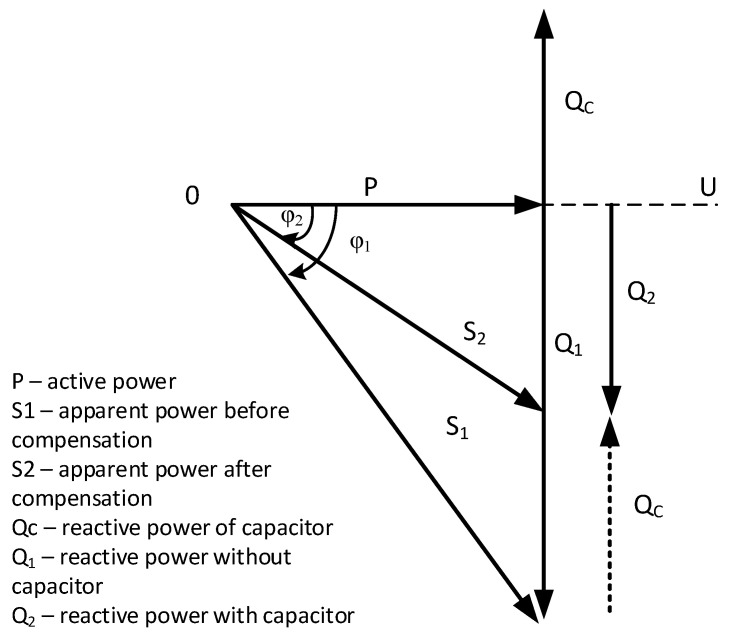
Vector diagram of power compensation.

In the case of machine tools, the best power factor control system will be using an individual compensation system based on a capacitor battery. A capacitor battery, consisting of capacitors of different capacitance connected in parallel, will cause the current to shift in phase concerning the voltage by 90°, thereby overtaking it. On the other hand, drives in machine tools cause a 90° shift (delay) of the current in phase to the voltage. According to the vector diagram of reactive power (inductive and capacitive), these powers compensate each other, resulting in a power vector with a lower value than before compensation (Figure 6).

The use of individual compensation is an advantageous solution in the case of using many consumers and in the case of expanding the plant with new machine tools without having to extend the existing power compensation system. An additional advantage of this type of compensation is the reduction of heat losses in all conductors by generating reactive energy at the point of consumption.

### 2.2. Experimental Setup and Data Acquisition

**Figure 7:**
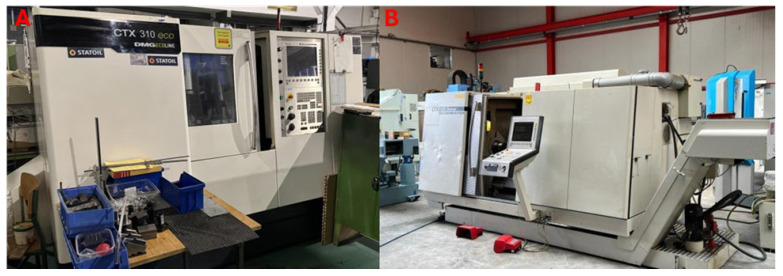
Investigated lathes: (**A**)—DMG CTX 310 Eco. (**B**)—CTX 420 Linear.

Two lathes characterized using different types of drives were selected for energy efficiency testing DMG CTX 310 eco (screw drive) and CTX 420 Linear (linear drive). These machine tools differed in their energy characteristics, which were mainly due to the electric drives used in them. The lathes are shown in Figure 7, and the parameters of the tested machines are described in Table 1.

In the context of numerically controlled machine tools, screw drives and linear drives are the most used drives. A screw drive typically comprises a motor, which is either synchronous or asynchronous, and a rolling-screw mechanism that enables the conversion of rotary motion into linear motion. A linear drive in opposition is based on a linear motor, which is a motor that has been developed into a linear form. This approach eliminates the need for a gearbox to change rotary motion into linear motion. This results in linear drives having a more compact design. Linear motors and ball screws are two standard drive systems for CNC lathes, each with distinct advantages. Linear motors offer higher precision, achieving positioning accuracy of 0.01 μm [57] and submicron accuracy [58]. They also provide better dynamic response and machining rates by eliminating mechanical issues associated with gears [59]. Ball screws, while less precise, offer high efficiency, load capacity, and good positioning accuracy. They can be optimized through proper controller settings to improve machining stability [60]. Linear motors excel in high-speed and high-precision applications, while ball screws are more cost-effective and flexible in configuration. Recent innovations, such as low-friction ball screw drawbars, have improved the efficiency and reduced power consumption of electromechanical actuators in CNC lathes [61]. Summarizing, the choice between screw drives and linear drives in CNC lathes involves careful consideration of the specific application requirements, including precision, speed, cost, and maintenance. While screw drives offer reliability and cost-effectiveness, linear drives provide superior speed and accuracy, albeit at a higher initial investment and complexity.

**Figure 8:**
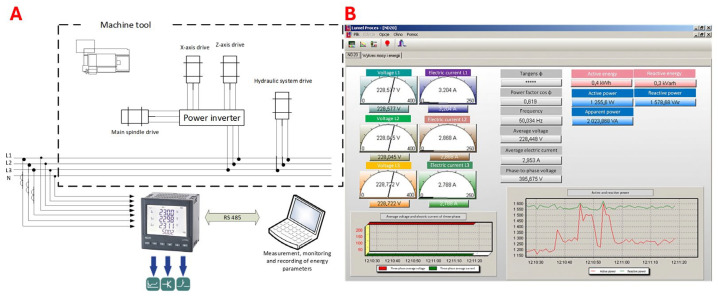
Wiring diagram of the power logging device (**A**) and program for recording the results of active and reactive power measurements on the machine tool “Lumel Process” (**B**). (***** asterisks next to the tangent fi indicate that the measurement was at the asymptote at a given moment, i.e. the fi angle was 90 degrees).

**Figure 9:**
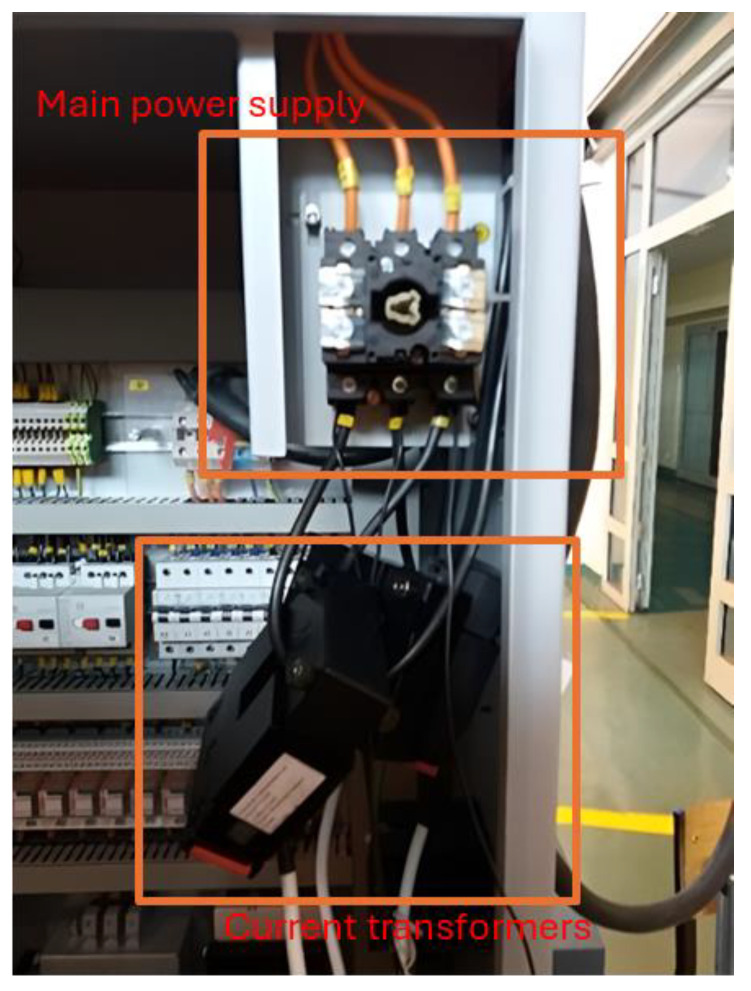
Measuring system with transformers mounted on the DMG CTX 310 Eco lathe main power supply.

Electricity consumption tests on the machine tool were carried out using a Lumel ND 20 logger (Lumel, Zielona Góra, Poland). The current was measured using 75 A current transformers with a current output 1 A, according to IEC60044-Accuracy Class 3, 0.5 VA (Lumel, Zielona Góra, Poland), while voltage was measured directly. The measurement was performed for both machine tools on the main power supply. The wiring diagram of the recorder is shown in Figure 8A. Recording the results was carried out using recorder software. An example window of the sheet created for recording is shown in Figure 8B, and the installed measurement system in the lathe control cabinet is shown in Figure 9.

**Figure 10:**
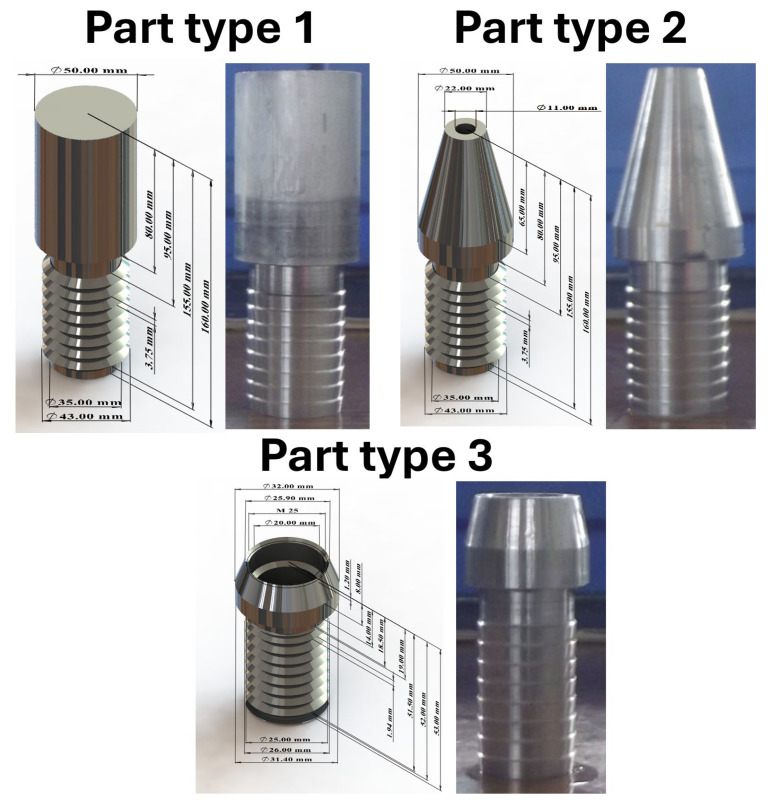
Parts prepared for turning on CTX 310 Eco.

**Figure 11:**
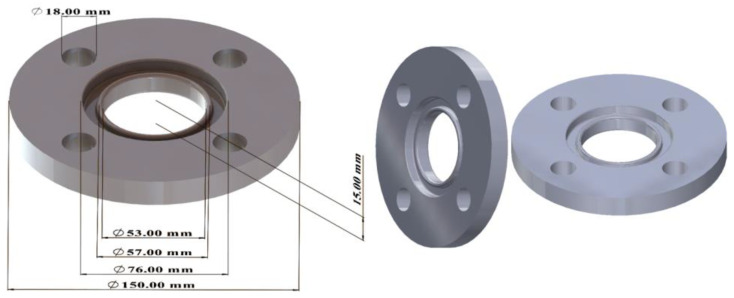
Parts prepared for turning on CTX 420 Linear.

Recording the measurement results took place in a 1 s time interval. Various products were machined during the study. Due to the impossibility of comparing the machining process of the same product on lathes, measurement results for machining and idling are presented. In addition, forced downtime still appears for the DMG CTX 310 Eco CNC lathe due to the nature of the machining process. The prepared parts for turning are shown in Figure 10 and Figure 11. The turned parts on the DMG CTX 310 Eco were made of aluminum rod, while those on the CTX 420 Linear were made of chrome–nickel steel.

## 3. Results and Discussion

### 3.1. Energy Calculations for Lathe with Screw Drives

The recorded results of measurements for the DMG CTX 310 Eco CNC lathe are shown in Figure 9, Figure 10 and Figure 11 and Table 2, Table 3 and Table 4. From the results obtained, the lathe under study has energy recovery systems during spindle deceleration, but this does not change the fact that when the active power obtained negative values, the value of the reactive power was the highest. The data are presented separately for idle run and processing/machining for all three parts, with the technological stop time distinguished. As a result, for all parts prepared for turning during machining/processing, the tanϕ value fluctuated from level 1.13 to 1.27. It was exceeded compared to the permissible value (tanϕ = 0.4) by nearly 200%. This high value translates directly into the electricity costs charged by operators.

**Figure 12:**
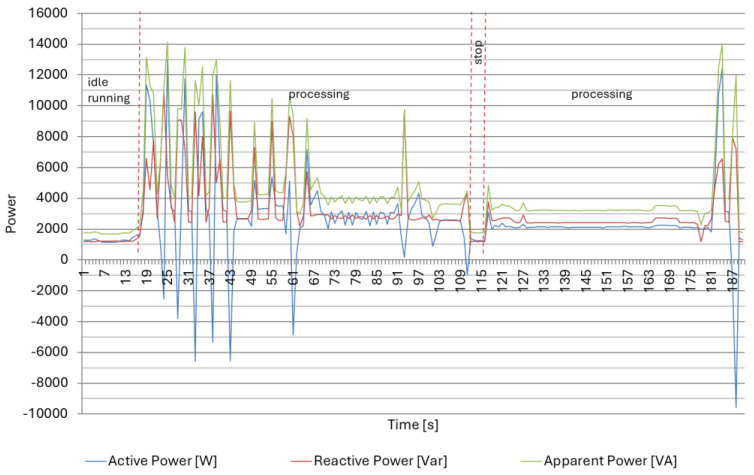
Power consumption by the CNC lathe DMG CTX 310 Eco in the selected time interval for Part type I.

As seen in Table 2 and Figure 12, during idle run and technological stop, active and reactive energy use were at a similar, stable level. The most significant use of active and reactive energy occurred during part processing. In addition, during processing, there were large fluctuations in active energy use, which is related to the energy return system during drive braking.

**Figure 13:**
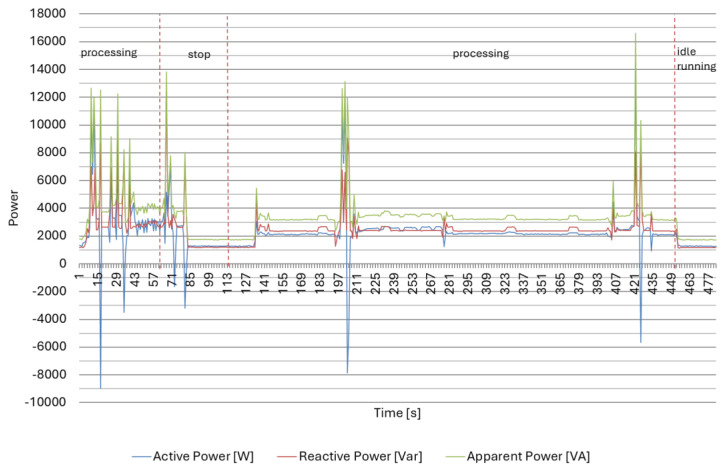
Power consumption by the CNC lathe DMG CTX 310 Eco in the selected time interval for part type II.

The measurement results for the production of part II in Table 3 and Figure 13 confirm the results obtained for part I. Similarly, the most significant use of active and reactive energy occurred during the machining of the part. Investigating the results of the interphase angle tanφ, we can see that its value decreased with a longer machining time.

**Figure 14:**
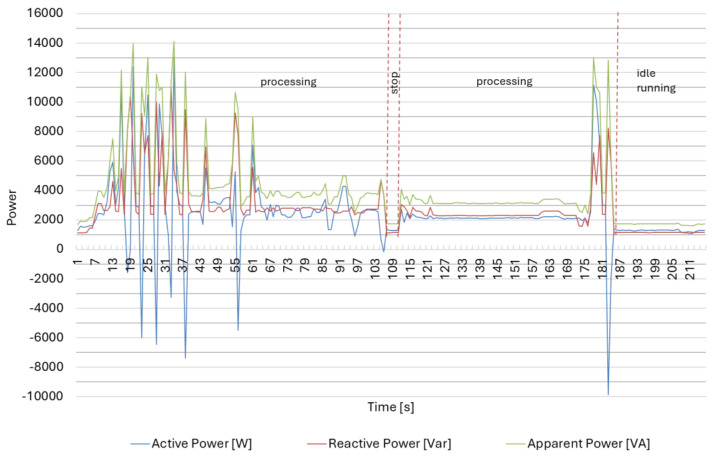
Power consumption by the CNC lathe DMG CTX 310 Eco in the selected time interval for Part type III.

The measurement results for the production of part III on a screw-drive lathe, presented in Table 4 and Figure 14, indicate a relationship between the processing time and the interfacial angle tanφ. This relationship suggests that the interphase shift angle tanφ value decreased as the processing time increased.

### 3.2. Energy Calculations for Lathe with Linear Drives

**Figure 15:**
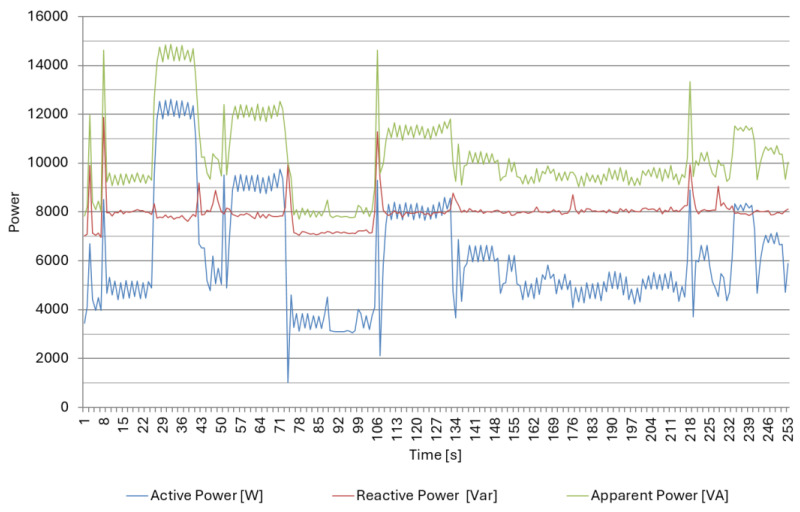
Power consumption by the CNC lathe DMG CTX 420 Linear in the selected time interval for machining of Part type IV.

**Figure 16:**
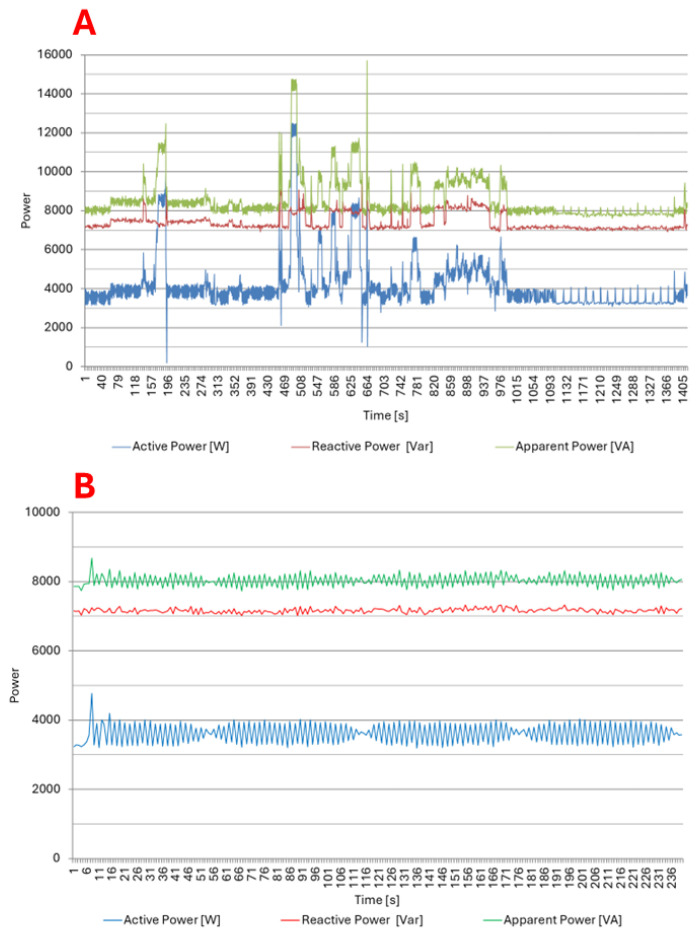
Power consumption by the CNC lathe DMG CTX 420 Linear in the selected time interval for setup time (**A**) and idle run (**B**) of Part type IV.

The measurement results for the CNC lathe DMG CTX 420 Linear are shown in Figure 15 and Figure 16 and Table 5. Due to the linear drives used, this lathe was characterized by significant reactive energy consumption already during an idle run (Figure 16B); the reactive power was at the level of 8 kVar and showed only slight changes of up to 20% during part processing (Figure 15). During the changeover (setup time), only a slight increase in active and reactive power consumption can be observed with the idle run. Active power consumption increased by 15%, while reactive power consumption increased by only 4%, which caused a decrease in the interphase angle’s tanφ to 1.78. From the observed results, it can be seen that during processing (Table 5), tanφ was at the level of 1.29, while during idle run, it reached the value of 1.98.

### 3.3. Compensation of Reactive Power

To determine the level of reduction of the use of reactive energy from the Operator’s network, we used Formulas (2) and (4) and corrected the interphase shift angle tanφ to the level of 0.4. Calculations were made for the system with compensation (Table 6).

**Figure 17:**
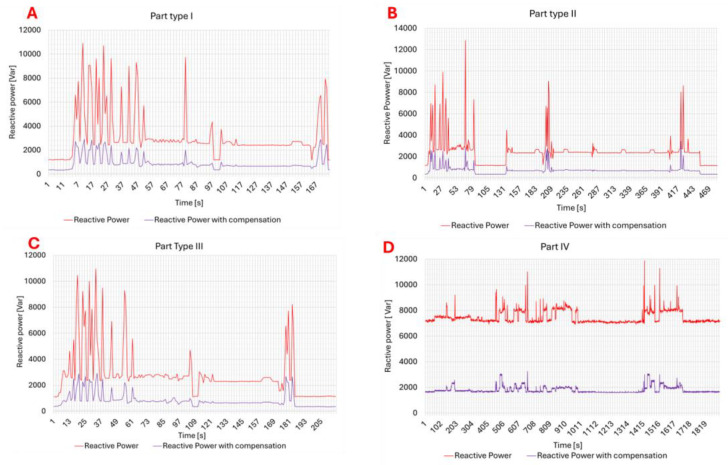
Reactive power consumption with and without compensation of CNC Lathe DMG CTX 310 Eco for (**A**) Part type I, (**B**) Part Type II, (**C**) Part Type III, and of CNC lathe DMG CTX 420 Linear (**D**) Part Type IV.

**Figure 18:**
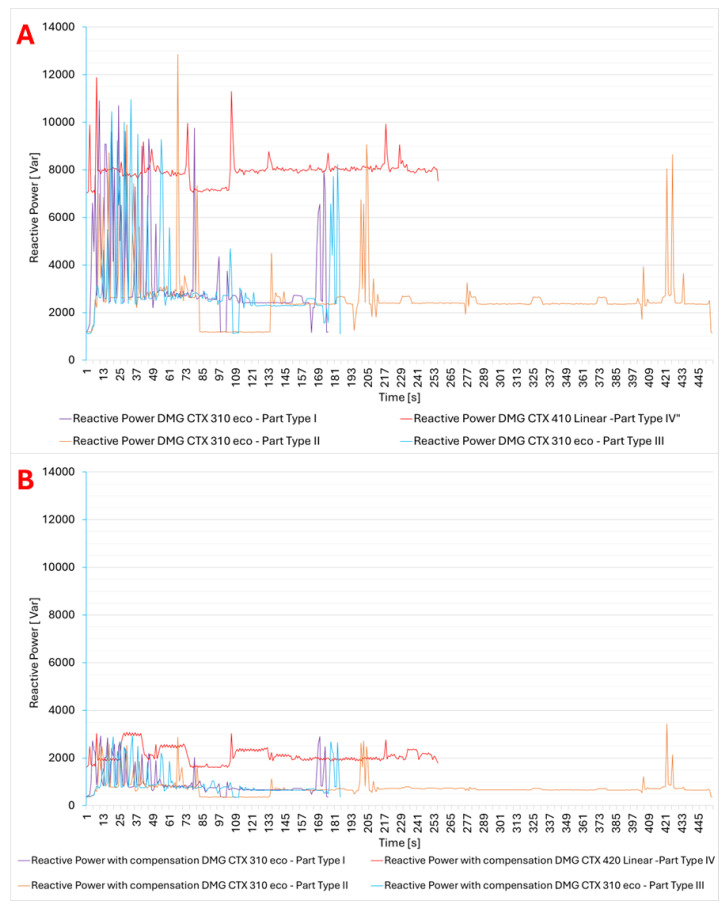
Reactive power consumption without compensation (**A**) and with compensation (**B**) during the machining time of CNC Lathe DMG CTX 310 Eco and CNC lathe DMG CTX 420 Linear.

The results of reactive power compensation for the production of individual parts, taking into account the type of lathe, are presented in Figure 17. The presented data show that for both lathes with screw drives (Figure 17A–C) and linear drives (Figure 17D), the reduction in reactive power consumption was almost 70%. In the case of lathes with screw drives, compensation allowed for a reduction in the amplitude of the fluctuation of the reactive power consumption. For machine tools with linear drives, individual compensation provided for a significant reduction in reactive energy consumption from the level of 8 kVar to the level of almost 2 kVar. Analyzing the reactive power consumption during the machining of parts for different products and types of lathes (Figure 18), the use of compensation allowed for power consumption at a level of 500–3500 Var as opposed to 1000–13000 Var for lathes without compensation.

Additionally, it can be seen that for lathes with linear drives, the reactive power consumption was more stable than in the case of lathes with screw drives. In addition, the use of compensation also reduces the amplitude of fluctuations. From the presented calculations (Table 6), we can conclude that the reduction in power use for lathes with screw drives was at the level of 23–30%, while for lathes with linear drives, it was at the level of 36–47%.

## 4. Conclusions

This study investigated reactive energy consumption in machine tools equipped with either screw or linear drives, revealing significant differences in their energy profiles. Our measurements on the DMG CTX 310 Eco (screw drives) and DMG CTX 420 Linear (linear drives) lathes demonstrated that linear drives consume substantially more reactive power, especially during the idle operation—a striking 130% higher than screw drives. Conversely, screw drives exhibited lower idle reactive energy consumption—around 200% less. During machining, both drive types showed comparable tanφ values (1.13–1.27 for screw drives and 1.29 for linear drives), suggesting that both can benefit similarly from reactive power compensation. Indeed, our results indicate that compensation can reduce reactive power consumption by nearly 70% for both drive types. However, compensation during idle operation is particularly crucial for linear-drive machine tools due to their high baseline consumption. We also observed that linear drives exhibited more stable reactive power consumption, with fluctuations within a narrow ±2 kVar range. Screw drives, in contrast, showed significant volatility of around 10 kVar. Compensation reduced these fluctuations in both cases, bringing them down to 500–3500 Var from 1000–13,000 Var without compensation. Moreover, a correlation between machining time and the interphase angle tanφ was identified specifically for screw-drive lathes, with tanφ decreasing as the machining time increased.

In summary, the application of reactive power compensation presents a significant opportunity to improve energy efficiency in both screw- and linear-drive machine tools. Compensation can lead to power consumption reductions of 23–30% for screw drives and up to 36–47% for linear drives. We summarize and outline the following:

- The lathe with linear drives consumed 130% more reactive power during idle operation compared to the lathe with screw drives.
- Screw drives showed significant fluctuations in reactive power (up to 10 kVar), while linear drives exhibited stable energy consumption with minor deviations of ±2 kVar.
- Reactive power compensation reduced energy consumption by nearly 70% for both lathe types.
- Energy savings achieved were 23–30% for screw drives and 36–47% for linear drives.
- For screw drives, there was a noticeable decrease in the interphase angle (tanϕ) with increased machining time.

## 5. Future Research

Future studies must further validate and expand the findings, considering the research results that were obtained. One key direction is to confirm the relationship between machining time and the interphase angle tanφ for lathes with linear drives. Next, research should focus on machining parts characterized by different machining times using a similar methodology to that used for lathes with screw drives. Additionally, to strengthen the conclusions, it is essential to verify the results by conducting experiments on the same type of part using identical machining and changeover times on both tested lathes. Another crucial aspect to explore is the impact of tool wear on energy consumption. As tools experience wear, their cutting efficiency changes, significantly affecting active and reactive power demands. Future research should investigate this relationship and aim to develop AI-powered tool condition monitoring systems. Such systems could predict optimal tool replacement times, minimizing energy waste and improving machining efficiency.

Furthermore, it is crucial to integrate energy monitoring and optimization strategies within the context of Industry 5.0, which emphasizes human–machine collaboration, data-driven smart manufacturing, and sustainability. Leveraging technologies like the Internet of Things (IoT), artificial intelligence (AI), and real-time data connectivity would enable more precise energy management and optimization of manufacturing processes. This integration would ensure the research findings are effectively implemented in modern manufacturing environments. Expanding the study scope to include digital twin technology could also provide valuable insights. Digital twins allow for virtual simulations of machining processes, enabling offline optimization of parameters and reducing experimental energy consumption and material waste. Finally, broader studies involving other machining processes, materials, and tools could identify universal energy-saving solutions applicable across diverse manufacturing settings. By pursuing these directions, future research can align with the goals of Industry 5.0 to develop energy-efficient, intelligent, and sustainable manufacturing solutions that address economic and environmental challenges.

## Figures and Tables

**Table 1:** Parameters of investigated lathes.

Machine Type	Unit	DMG CTX 310 Eco	CTX 420 Linear
Main Spindle			
Power output (40%/100% DC)	KW (AC)	16.5/11	45/36
Max. torque (40%/100% DC)	Nm	166.5/112	750/600
Max. Rotational speed	rpm	5000	4000
Tool holder			
Power output (40% DC)	kW	8.4	10.6
Max. torque (40% DC)	Nm	20	22.5
Max. rotational speed	rpm	4500	5000

**Table 2:** Measurement results for Part type I by type of work for the DMG CTX 310 Eco machine.

Part Type	Time [s]	Operation	Active Power [kW]	Reactive Power [kVar]	Apparent Power [kVA]	Active Energy [kWh]	Reactive Energy [kVarh]	tanϕ
Part type I	172	Processing	2.67	3.38	4.74	0.1275	0.1613	1.2650
	4	Stop	1.27	1.19	1.74	0.0014	0.0013	0.9433
	14	Idle running	1.23	1.21	1.74	0.0048	0.0047	0.9818

**Table 3:** Measurement results for Part type II by type of work for the DMG CTX 310 Eco machine.

Part Type	Time [s]	Operation	Active Power [kW]	Reactive Power [kVar]	Apparent Power [kVA]	Active Energy [kWh]	Reactive Energy [kVarh]	tanϕ
Part type II	406	Processing	2.40	2.69	3.75	0.2683	0.3031	1.1296
	49	Stop	1.27	1.19	1.75	0.0173	0.0162	0.9331
	27	Idle running	1.19	1.17	1.68	0.0119	0.0117	0.9835

**Table 4:** Measurement results for Part type III by type of work for the DMG CTX 310 Eco machine.

Part Type	Time [s]	Operation	Active Power [kW]	Reactive Power [kVar]	Apparent Power [kVA]	Active Energy [kWh]	Reactive Energy [kVarh]	tanϕ
Part type III	181	Processing	2.52	3.14	4.44	0.127	0.150	1.1811
	4	Stop	1.28	1.93	2.71	0.001	0.001	0.8940
	31	Idle running	1.27	2.34	3.33	0.011	0.010	0.9077

**Table 5:** Measurement results for Part type III by type of work for the DMG CTX 420 Linear lathe.

Part Type	Time [s]	Operation	Active Power [kW]	Reactive Power [kVar]	Apparent Power [kVA]	Active Energy [kWh]	Reactive Energy [kVarh]	tanϕ
Part type IV	256	Processing	6.15	7.96	10.20	0.437	0.566	1.2932
	1415	Setup time	4.17	7.42	8.57	1.638	2.915	1.7796
	240	Idle running	3.62	7.16	8.03	0.241	0.478	1.9811

**Table 6:** Savings possible thanks to reactive power compensation.

Lathe		CNC DMG CTX 310 Eco									CNC CTX 520 Linear		
Part Type		I			II			III			IV		
Operation		processing	stop	idle running	processing	stop	idle running	processing	stop	idle running	processing	setup	idle running
Time, s		172	4	14	406	49	27	181	4	31	256	1415	60
tanφ		1.27	0.94	0.98	1.13	0.93	0.98	1.18	0.89	0.91	1.29	1.78	1.98
tanφ with Possible Compensation		0.38	0.38	0.38	0.38	0.38	0.38	0.38	0.38	0.38	0.38	0.38	0.38
Consuming Active Power, kWh	without compensation	2.67	1.27	1.23	2.40	1.27	1.27	2.52	1.28	1.27	6.15	4.17	3.62
	possible to achieve with individual compensation	2.67	1.27	1.23	2.40	1.27	1.27	2.52	1.28	1.27	6.15	4.17	3.62
Consuming Reactive Power, Varh	without compensation	3.38	1.19	1.21	2.69	1.19	1.17	3.14	1.93	2.34	7.96	7.42	7.52
	possible to achieve with individual compensation	0.98	0.36	0.36	0.78	0.36	0.36	0.92	0.36	0.36	2.11	1.84	1.77
Consuming Apparent Power, KVAh	without compensation	4.74	1.74	1.74	3.76	1.75	1.73	4.44	1.73	1.72	10.20	8.57	8.90
	possible to achieve with individual compensation	3.32	1.32	1.29	2.70	1.32	1.32	3.15	1.33	1.32	6.52	4.54	4.98
% Of Energy Reduction		30%	24%	26%	28%	24%	24%	29%	23%	23%	36%	47%	44%

## Data Availability

The datasets presented in this article are not readily available due to technical limitations. Requests to access the datasets should be directed to corresponding author.
